# A magnetic affinity approach to identify plant GABA-binding proteins

**DOI:** 10.3906/biy-1901-79

**Published:** 2019-08-05

**Authors:** Jie ZOU, Jingzhe GUO, Shisheng LI

**Affiliations:** 1 Guiyang College of Traditional Chinese Medicine, Guiyang, China; 2 Key Laboratories of Economic Forest Germplasm Improvement and Comprehensive Resources Utilization of Hubei Province, College of Life Science, Huanggang Normal University, Huanggang, China; 3 College of Life Science, State Key Laboratory of Plant Hybrid Rice, Wuhan University, Wuhan, China

**Keywords:** GABA, receptor, magnetic bead, signaling, pollen

## Abstract

In plants, GABA plays a critical role in sexual plant reproduction; however, GABA receptors and the associated detailed signaling mechanisms remain to be elucidated. Our experiments show that the proposed technique is reliable and convenient for probing GABA-binding proteins and could be applicable in similar projects by covalently immobilizing the free carboxylic group of GABA on magnetic beads (SiMAG-Carboxyl). New probes produced by covalently immobilizing the free carboxylic group of GABA on magnetic beads (SiMAG-Carboxyl) can obtain useful information on GABA receptors in plants.

## 1. Introduction

γ-Aminobutyric acid (GABA), a four-carbon, nonprotein, conserved amino acid, is found in various species, from the simplest bacterium to higher organisms (Bouché and Fromm, 2004; Podlesakova et al., 2019). In mammals, GABA is present at high concentrations in the central nervous system and is a primary inhibitory neurotransmitter. It activates various signaling pathways by binding to several types of receptors and anchoring them to the membranes of neural cells (Bormann, 2000; Bettler et al., 2004). In addition, GABA is a significant component of the free amino acid pool in plants and it is present in almost all organs. The intercellular GABA content can be increased quickly in response to biotic and abiotic stresses, such as cold, drought, salinity, extreme temperatures, oxidative stress, and fungal infections (Bouche et al., 2003; Jiang and Gu, 2003; Jalil et al., 2017). It has been postulated that GABA is an endogenous plant signaling molecule (Palanivelu et al., 2003; Yang, 2003). Recently, it was shown that GABA regulates anion transport via aluminum-activated malate transporter (ALMT) proteins in various plants (Ramesh et al., 2017). ALMT was the first transporter protein found to be regulated by GABA in plants. This discovery rendered ALMT a prime candidate for mediating GABA-based signaling in plants and provided new impetus for exploring the regulation of the potential interactions between GABA and other signaling molecules. However, little is known of how the GABA-ALMT model regulates plant development and growth, especially in terms of whether and how GABA functions as a signaling molecule in the style and mediates interactions with the pollen tube. Are there other GABA receptors in plants? What are the components of the “GABA signaling complex”? These questions still need to be answered.We previously identified GABA-binding sites on tobacco mesophyll and pollen protoplasts, and the binding of GABA to the plasma membrane triggered calcium signaling (Yu, 2006). GABA-related microarray data showed that exogenous GABA mediates the expression of certain plant genes (Lancien and Roberts, 2006). All of these results support the hypothesis that GABA receptors exist on plant plasma membranes acting as transducers for GABA signaling. Therefore, it would be interesting to confirm the existence of these receptors and compare them with their counterparts in animals. However, BLAST searches of the GABA receptors (GABAA,B,C receptors) expressed in animals failed to identify equivalent homologs in plant cells (Lacombe, 2001); therefore, unique approaches are needed to identify GABA receptors or their binding proteins on plant plasma membranes.In animals, cDNA of the first GABAB receptor was cloned into rats using the high-affinity antagonist CGP64213 as a hybridization probe. In these experiments, mammalian GABAB receptors were shown to be G-protein-coupled receptors that repressed Ca2+ influx by activating heterotrimeric G-proteins (Mintz and Bean, 1993; Kaupmann, 1997). Therefore, we followed the techniques used in animals to design a simple but useful activity-based fluorescent probe linked to CGP64213 to search for GABAB-like receptors expressed on the plant plasma membrane. Our results showed that the technique used in animals was not suitable in plants because the plant GABAB receptor, if it exists, is different from its animal counterpart in both sequence and binding-site spatial structure. We used biomagnetic techniques to separate GABA-binding proteins because these techniques are fast and simple and can deal with large sample volumes without the need for time-consuming centrifugation steps. We covalently immobilized the free carboxylic group of GABA on magnetic beads (SiMAG-Carboxyl) to generate GABA-linked magnetic beads and attempted to purify GABA-binding proteins from the total protein of Arabidopsis. The fractions collected from the GABA-SiMAG-Carboxyl beads were washed with a buffer containing high concentrations of GABA and NaCl and then separated by SDS-PAGE. After tandem mass spectrometry (MS/MS) analysis, we successfully identified several candidate GABA-binding proteins in Arabidopsis. This work showed that our protocol was useful and convenient for probing unknown receptors on the plant cell surface.

## 2. Materials and methods

### 2.1. Probes used for animal cells

The CGP64213-coupled probes (probeR and probeC) were kindly provided by Professor Jian-Feng Liu at Huazhong University of Science and Technology. All chemicals were purchased from Sigma unless otherwise stated.

### 2.2. Plant material and growth conditions

*Nicotiana tabacum* SR1 plants were grown at 23 °C in a greenhouse at Wuhan University, China, under a 16-h photoperiod. Arabidopsis (*Arabidopsis thaliana* Col-0) plants were grown in a 1:1:1 mixture of vermiculite, perlite, and peat moss in an environmentally controlled chamber with a long photoperiod (16-h light and 8-h dark) at 22 °C.2.3. Tobacco pollen and mesophyll protoplasts isolationTobacco pollen protoplasts were isolated as follows. The pollen grains were germinated in germination medium [5 µmol CaCl2, 5 µM Ca(NO3)2, 1 mM Mg(SO4)2, 0.01% H3BO3, and 18% sucrose, PH 6.5-7.0] at 25 °C. When the pollen tubes emerged from the germ pore, the pollen was transferred into an enzyme solution [1% cellulose (Onozuka R-10) and 1% pectinase (Yakult), dissolved in basic solution containing 1 M mannitol, 0.4 M sorbitol, 3 mM KNO3, 50 mM CaCl2.2H2O], and incubated at 28 °C for 1 h to release the pollen protoplasts. Tobacco mesophyll protoplasts were isolated by stripping away the epidermal layer of young tender leaves and cutting them into 0.5-1 mm leaf strips with fresh razor blades. These were immersed in enzyme solution (0.5% cellulose R-10, 0.6% pectinase, 9% mannitol, pH 5.8) in Petri dishes at 25 °C for 1 h. The released protoplasts were collected manually and washed for later use.

### 2.4. Labeling rat brain tissue sections and protoplasts using photoaffinity probes

Frozen sections of the rat hippocampus were kindly provided by Professor Xiao-Dong Li of Wuhan University. The frozen sections were incubated with 1 μM probeR or probeC in phosphate-buffered saline (PBS) for 30 min at 37 °C and then irradiated under an ultraviolet (UV) lamp (365 nm) for 15 min at room temperature (RT). After two washes with PBS, the sections were observed under an Olympus FV1000 microscope at RT.The collected pollen and mesophyll protoplasts were incubated with 1 μM of the same probes in the basic solution (protoplast isolation medium without the enzyme) for 1 h at 25 °C, in darkness. Then, they were irradiated by UV (365 nm) for 10 min at RT. After two washes with the basic solution, the protoplasts were observed under a microscope at RT.

### 2.5. Determination of total protein content

First, 10-40 g of fresh Arabidopsis tissues (including leaf and flower tissue) were ground into a powder and transferred into an 10-mL tube containing ice-cooled protein extraction buffer (10 mM MES, 100 mM MgCl2, 2 mM EGTA, 2 mM 1,4-dithiothreitol, 1 mM phenylmethylsulfonyl fluoride, 0.5% (v/v) Triton X-100 and 1× protein inhibitor cocktail [Complete™; Roche]). The sample was extracted for 3 h on ice, and then the homogenate was centrifuged for 15 min at 15,000 × *g*. The supernatant was transferred into a new tube and centrifuged again at 100,000 × *g* for 30 min. Finally, the supernatant was collected, and the extract was condensed using Amicon Ultra filters (Millipore). The protein concentration of the condensed extract was determined using a Bio-Rad Protein Assay Kit based on the Bradford method. To maintain the native protein form throughout the experiment, the protein extracts were kept at 0-4 °C and no ionic detergents were added.

### 2.6. The synthesis of GABA linked affinity magnetic beads

The SiMAG-Carboxyl (0.5 µm ϕ, Chemicell. Prod. No. 1402) particles were washed twice with 1 M MES buffer using a magnetic separator (Promega). After the second wash, the magnetic particles were resuspended in MES buffer containing 10 mg EDC (Thermo Fisher). Subsequently, only freshly prepared EDC was added to the particles, followed by mixing on a shaker for 10 min at RT. After this step, the EDC solution was removed and replaced by prepared NHS (Thermo Fisher), followed by fully mixing on a shaker and reaction at RT for 30 min. Then, the NHS was removed and an amine group containing ligand GABA (10 mg dissolved in double distilled water) was added to the activated particles and mixed on a shaker for 2-3 h at RT. Next, the particles were resuspended in blocking buffer (10 mm hydroxylamine [Thermo Fisher]), shaken, and reacted for 10 min at RT to terminate the reaction. Finally, the particles were resuspended in PBS for storage and later use.

### 2.7. Magnetic affinity chromatography and interactome identification

All steps were performed at 4 °C. The GABA-linked magnetic beads were resuspended, removed using the magnetic separator (Promega), and then added to 1 mL MES protein extraction buffer and preequilibrated by incubating on a rotator at 50 rpm at 4 °C for 1 h. The MES buffer was then removed from the beads using the magnetic separator. About 200 µL of preequilibrated GABA-linked magnetic beads were incubated with 1.2 mL condensed Arabidopsis total protein extract on a rotator at 50 rpm at 4 °C for 4-6 h. The total protein plus 100 mM GABA were applied in the control group. Then, the supernatant was carefully removed and 1 mL MES buffer plus 100 mM NaCl were added to the protein-bound magnetic beads to wash off nonspecifically bound proteins on the beads. The supernatant was removed again, and the beads were washed with 1 mL MES buffer plus 200 mM NaCl. The binding interactome from the bead was eluted using a high-concentration salt and GABA competitive elution (MES buffer plus 200 mM NaCl and 100 mM GABA), and the eluted fractions were collected and condensed for subsequent analysis.The proteins were separated using one-dimensional sodium dodecyl sulfate-polyacrylamide gel electrophoresis (SDS-PAGE) according to the molecular cloning protocol. After SDS-PAGE, the gel was rehydrated in ultrapure water for 30 min; this step was repeated twice with fresh water. Using a clean scalpel, the entire target band for in-gel tryptic digest (IGTD) was excised according to Huynh et al. (2009). The trypsin-digested peptides were analyzed by matrix-assisted laser desorption/ionization time-of-flight mass spectroscopy (MALDI-TOF-MS) following the standard procedures of the proteomics facility to which the samples had been submitted. Monoisotopic masses from all spectra recorded for a given peptide were selected and further analyzed. The experimental mass spectra were matched with theoretical mass spectra obtained from various Arabidopsis databases using a sequence database and confirmed using the Bradford assay (Bio-Rad).

## 3. Results

### 3.1. Probe characterization

**Figure 1:**
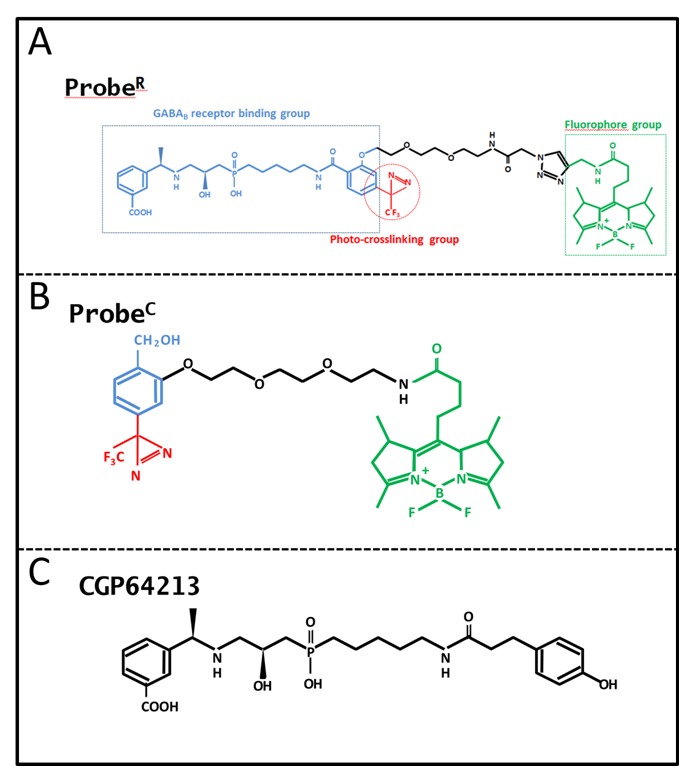
The characterization of the probeR, probeC, and CGP64213. (A) The structure of probeR. The probeR comprises GABAB receptor binding group CGP64213 (outlined in blue), a photolabile diazirine group (outlined in red), a 4,4-diflupro-4-bora-3a, 4a-diazas-indacene (BODIPY) as the fluorophore (outlined in green). (B) The structure of probeC. ProbeC is a probe with only photolabile diazirine group (outlined in red) and BODIPY fluorophore (outlined in green). (C) The structure of GABAB receptor selective high-affinity antagonist CGP64213; probeR and probeC were designed based on CGP64213.

To determine if the techniques used in animals can be used to probe plant GABAB receptors, probeR was synthesized. This probe was successfully applied in photo-affinity labeling of GABAB receptors transiently expressed in CHO cells (Li et al., 2008). ProbeR showed specific binding to the GABAB subunits and high specificity in photo-affinity labeling. It was suitable for localizing GABAB receptors on living mammalian cells. ProbeR was designed based on CGP64213, a high-affinity antagonist of GABAB receptor (Figures 1A and 1B). In addition to its receptor-binding domain, the probe also contains a photolabile diazirine group that effectively generates an irreversible covalent linkage between the probe and target receptor after UV-irradiation, and the fluorescent reporter dye BODIPY (boron-dipyrromethene; 4,4-difluoro-4-bora-3a, 4a-diaza-s-indacene), which is used to observe the localization of labeled receptor proteins. Photo-induced cross-interaction between the probe and the targeted receptor was used to increase the labeling stability. BODIPY is a fluorophore with fascinating spectral characteristics; it possesses a high environment-independent fluorescence quantum yield, making it an important tool in a variety of imaging applications (Karolin et al., 1994). Meanwhile, we designed another control probe called probeC, which does not possess the antagonist group CGP64213 and only contains the fluorescence and photo-affinity groups (Figure  1C).

### 3.2. Imaging of GABA-binding sites using probes

**Figure 2:**
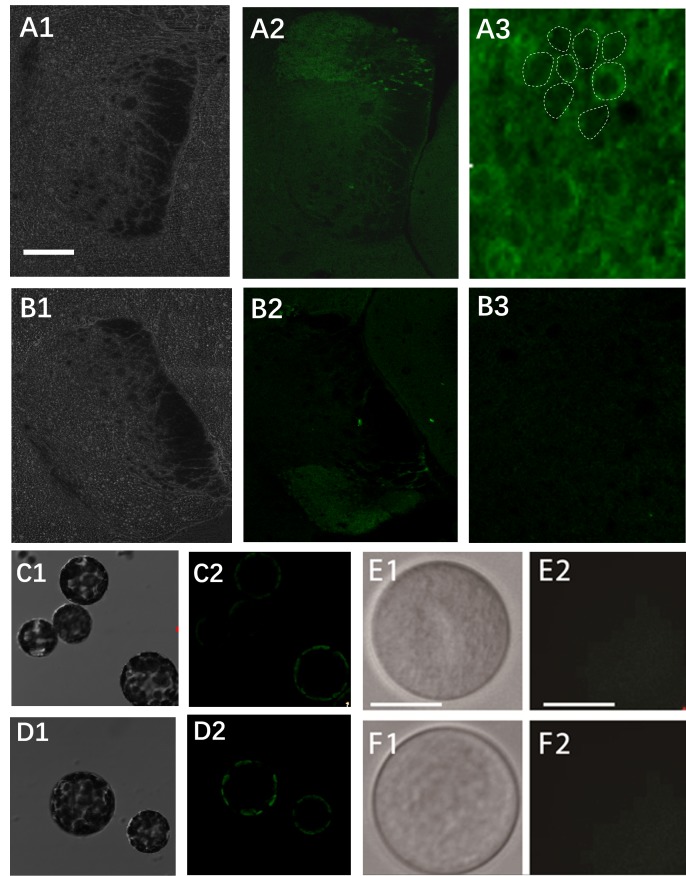
Detection of the GABA binding sites on the rat brain sections, tobacco mesophyll protoplasts, and pollen protoplasts. (A1) Bright field view of rat brain section incubated with probeR; (A2) CLSM image of the same tissue section as in A1; (A3) partially enlarged image of the same section in A2; the dotted lines indicate the circular green fluorescence around the Hippocampus zone cells; (B1) Bright field view of rat brain section incubated with probeC; (B2) CLSM image of the same section as in B1; (B3) partially enlarged image of the same section as in B2; (C1) Bright field view of tobacco mesophyll protoplasts incubated with probeR; (C2) CLSM image of the same section as in C1; (D1) Bright field view of tobacco mesophyll protoplasts incubated with probeC; (D2) CLSM image of the same section as in D1; (E1) Bright field view of tobacco pollen protoplasts incubated with probeR; (E2) CLSM image of the same section in E1; (F1) Bright field view of tobacco mesophyll protoplasts incubated with probeC; (F2) CLSM image of the same tissue section as in F1; The scale bar is 20 µm.

Before the labeling studies in plant protoplasts, the probes were first evaluated for their ability to label GABAB receptors in rat brain tissue sections in situ labeling studies. As shown in Figure  2green fluorescence was abundantly detected in the hippocampal zone (Figures 2A1-2A3), and a circular ring of fluorescence was observed in the hippocampal zone (Figure  2A3, enlarged area). This was consistent with in situ hybridization results obtained from a previous work (Carter and Mynlieff, 2004; Omar, 2009). Control probeC-treated sections showed no fluorescence (Figures 2B1-2B3), indicating that the GABAB receptors on the membranes of hippocampal zone cells were specifically labeled.Since the probe was feasible for labeling GABAB receptors, we further tested whether the probe could bind to similar GABA receptors on the surface of plant protoplasts. Mature mesophyll protoplasts were isolated according to a previously published protocol (Iwahara et al., 1998); mature pollen protoplasts were also isolated using a germination-enzymatic treatment protocol established in our lab (Yu et al., 2006). Both types of living protoplasts were isolated, collected, and incubated with the probes. As shown in Figures 2C1-2F2, a circle of chloroplast autofluorescence was observed close to the membrane of the mesophyll protoplasts (Figure  2C2). A similar pattern was also observed in the probeC-treated control group (Figure  2D2). At the same time, the same probes were also applied to pollen protoplasts, but no green fluorescence was observed after treatment with probeR or probeC (Figures 2E1-2F2). These results indicate that the probe that specifically bound to GABAB receptors in animal cells cannot bind to GABA receptors or their binding proteins in plant cells.

### 3.3. Synthesis and characterization of GABA-coupled magnetic nanoparticles

**Figure 3:**
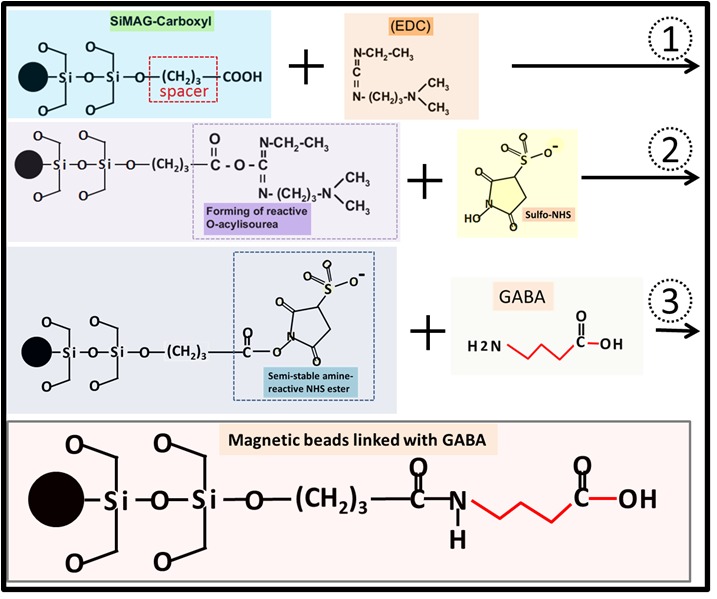
Schematic diagram showing the preparation of GABA-linked magnetic beads. 1. The SiMAG-Carboxyl is reacted with EDC and forms a reactive O-acylisourea end; 2. The reactive O-acylisourea end of SiMAG beads reacts with sulfo-NHS and forms a semi-stable amine reactive NHS ester end; 3. The semi-stable amine reactive NHS ester end reacts with the amine group of GABA and forms the GABA linked SiMAG beads.

**Figure 4:**
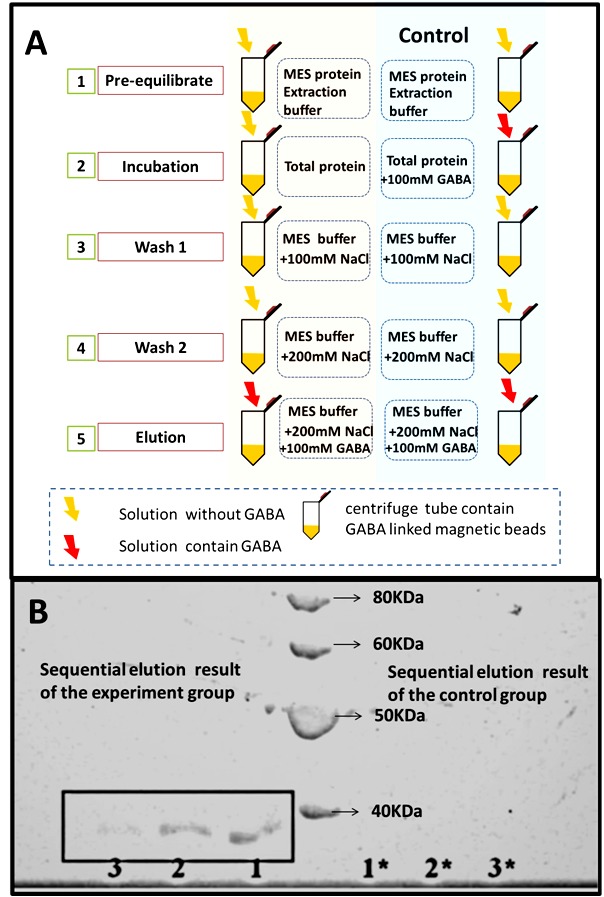
Schematic procedure of the magnetic affinity purification and the SDS-PAGE result of sequential elution fractions.****(A) The complete process of magnetic affinity purification of the GABA binding proteins; (B) the SDS-PAGE result of sequential elution (100 Mm, 200 mM, 300 mM NaCl plus 100 mM GABA) fractions of experiment group (1, 2, 3), and control group (1*, 2*, 3*).

GABA was combined with quantum dots (QDs) through the formation of amide linkages between the carboxylate group of QDs and the amino group of GABA. GABA-QDs can bind to tobacco protoplast membranes (Yu, 2006). We then chose GABA as the ligand and SiMAG-Carboxyl magnetic beads as the matrix to trap the binding partners in plant cells. SiMAG-Carboxyl beads contain a three-atom spacer arm, to which a free carboxylate group is conjugated. To link GABA to the magnetic beads, carbodiimide was used to mediate the formation of amide linkages between GABA and SiMAG-Carboxyl. Carbodiimides react with the terminal carboxylate groups of SiMAG-Carboxyl to highly reactive O-acylisourea derivatives and react readily with amino groups from GABA. These reactions require the crosslinking reagent ethyl-3-(dimethylaminopropyl) carbodiimide and the catalyst N-hydroxysuccinimide (Figure  3).3.4. Affinity purification and identification of GABA-binding proteinsThe novel affinity chromatography matrix designed above (GABA-SiMAG) was used to isolate GABA receptors and binding proteins from the total proteins of Arabidopsis. The extracted and condensed total proteins were incubated with the GABA-SiMAG beads. As a negative control, total proteins and GABA (100 mM) were incubated with the GABA-SiMAG beads. A buffer with a high concentration of GABA competitively bound to GABA-binding proteins among the total proteins, resulting in elution of GABA-binding proteins from the exposed GABA groups of the magnetic beads. Then, sequential elution was applied to remove low-affinity and nonspecifically bound proteins from the GABA-SiMAG, resulting in enrichment of specific and high-affinity GABA-binding proteins bound to the magnetic beads. A final elution buffer containing 200 mM NaCl with 100 mM GABA was used to elute specific GABA-binding proteins competitively (Figure  4A). SDS-PAGE of the final eluted fractions is shown in Figure  4B. The purified GABA-binding proteins migrated as a single protein band on SDS-PAGE, showing an estimated molecular mass of 40 kDa (Figure  4B). Proteins that bind to the GABA group of GABA-SiMAG beads with high affinity form the GABA interactome. To identify the affinity-purified proteins, a standard proteomics approach was employed. The entire protein band was excised and subjected to IGTD. Finally, the recycled peptides were analyzed by MS/MS for de novo sequencing. The Table shows the sequencing results. Eighteen proteins were identified as putative GABA interactome proteins; most were proteins encoded by housekeeping genes, including actin, heat shock protein, elongation factors, histone, and chaperonin. These proteins are strongly expressed in plants and are the most abundant proteins in cells. Therefore, we believe that these proteins are nonspecific binding components on magnetic beads. We focused on three proteins: beta glycosidases, ABCG transporters, and the U-Box 44 protein. Beta glycosidases are involved in beta-glucan synthesis during cell wall development and in defense mechanisms, and belong to the glycosyl hydrolase family; they catalyze the hydrolysis of glycoside bonds. The glycosidase obtained in this experiment was myrosinase, which catalyzes the hydrolysis of glucosinolates into compounds that are toxic to various microbes and herbivores. In Arabidopsis, myrosinase catalyzes the hydrolysis of glucosinolate to sulforaphane, which is specific to cruciferous plants and has anticancer effects. Interestingly, the skeletons of GABA and sulforaphane are similar. The relationship between GABA and myrosinase should be explored, and whether GABA can affect the catalytic reaction of myrosinase should be determined (Davies., 1995; Chun et al., 2018). ABCG transporters belong to the ATP-binding cassette (ABC) transporter family and contribute to many fundamental processes, such as phytohormone transport, surface lipid deposition, and plant-microbe interactions (Rea, 2007; Kretzschmar et al., 2011). The GABA transporter *Bar*,**which is found in *Rhizobium leguminosarum*, also belongs to the ABC transporter family (White and Prell, 2009). The U-Box 44 protein functions as an E3 ubiquitin-protein ligase and primarily prevents premature senescence by targeting proteins for degradation. The phytohormone-like auxin receptor TIR, gibberellin receptor GID1, and jasmonic acid receptor CO1 are ubiquitin-protein ligases that contain an F-box domain (Moon et al., 2004; Dharmasiri et al., 2005; Yan et al., 2009). We tried to express and purify these proteins, but unfortunately they are very difficult to express in different systems using traditional methods.

**Table 1:** The MS/MS result of the purified single band.

Number	TAIR accession	Description
P1	AT5G42020	Luminal-binding protein 2
P2	AT5G26000	Beta glucosidase 38, TGG1
P3	AT5G02500	Heat shock cognate 70 kDa protein 1
P4	AT2G37620	Arabidopsis Actin-1
P5	AT2G28000	RuBisCO large subunit-binding protein subunit alpha
P6	AT2G33210	Chaperonin CPN60-like 1
P7	AT1G55490	RuBisCO large subunit-binding protein subunit beta
P8	AT4G24190	HSP90-like protein
P9	AT1G07660	Histone H4
P10	AT1G51060	Probable histone H2A.1
P11	AT4G02930	GTP binding Elongation factor Tu family protein
P12	AT3G13860	HEAT SHOCK PROTEIN 60–3A, HSP60–3A
P13	AT1G63250	DEA(D/H)-box RNA helicase family protein
P14	AT1G51460	ABC transporter G family member 13
P15	AT3G14210	Epithiospecifier modify1 ESM1
P16	AT5G59370	Arabidopsis Actin-4
P17	AT1G07940	GTP binding Elongation factor Tu family protein
P18	AT1G20780	U-box domain-containing protein 44 Arabidopsis thaliana

## 4. Discussion

GABA is a neurotransmitter in animals that has been studied extensively. Different types of GABA receptors are distributed on cell surfaces and play critical roles in GABA signaling (Luján, 2007; Olsen and Sieghart, 2008). The techniques used to identify these receptors are based mainly on the design of specific probes found to be very useful for receptor localization and characterization (Froestl, 1999).In plants, GABA acts in response to a variety of environmental signals, including hypoxia, acidosis, mechanical stress, and cold stress (Baum, 1996; Shelp et al., 1999; Renault, 2011). In Arabidopsis, GABA plays an essential role in pollen tube growth (Palanivelu, 2003). Our previous work revealed that GABA binds to the plasma membranes of both pollen and somatic cell protoplasts and triggers calcium influx (Yu, 2006). We thus proposed that there may be similar GABA receptors on the membrane surface of plant cells that function similarly to their animal counterparts. Based on our observations in plant cells, we hypothesized that the GABAB type receptor most likely exists in plant cells. Therefore, we tried to identify gene and protein homologs of the animal GABAB receptor in plants by database searches. Unfortunately, no such homologs could be found. However, based on previous experience, there may be some proteins that differ from the GABAB receptor in their amino sequence but have similar spatial structure in their binding sites. Such proteins, therefore, could also bind to GABA and function in a similar way. Thus, although a homolog of the animal GABAB receptor was not found in plants, it is necessary to confirm whether there are indeed proteins capable of binding to GABA and functioning as GABA receptors via similarities in spatial structure. Therefore, we evaluated reliable probes used in animal cells and confirmed that they cannot bind to plant cell membranes. These results showed that if GABA receptors or binding proteins do exist on plant cell membranes, they are different from their animal counterparts in both sequence and binding-site spatial structure.To identify GABA receptors on plant cell membranes, we developed a unique technique. As described above, we synthesized GABA-linked affinity magnetic beads. We designed a spacer between the coated magnetic bead and GABA molecule that allows GABA to expand its spatial structure fully to bind potential receptors or any other binding proteins. Because of the magnetic beads, this probe was quite efficient in repeated experiments. Although the candidate receptors have not yet been fully analyzed, since we need to confirm each protein individually, these results indicate that the technique has great potential for similar use in finding unknown binding proteins. In particular, this technique may be combined with QDs that were used in our previous work (Yu, 2006) for identifying receptors on plant cell membranes. Another advantage of this probe is that it can be easily used to purify binding proteins. As previous experiments on protein isolation have shown, the proteins bound to affinity magnetic beads are easily eluted (Ivo and Mirka, 2011). Therefore, this is a convenient alternative technique for purifying directly and indirectly bound proteins and can be used in many similar experiments.Although ALMT, a transporter directly regulated by GABA, has been found in plants (Ramesh et al., 2015), in order to understand the GABA signaling pathway it is necessary to explore the components directly bound to GABA, or present in GABA signaling complexes in plants. We used affinity chromatography and GABA as “bait” to try to catch GABA-binding proteins. The key to affinity chromatography lies in the ability of the bait to bind to the receptor. Therefore we have also tried to use GABA receptor agonists in animals as bait. Unfortunately, CGP64213 cannot bind to GABA receptors on the surface of plant cells, so we had to use GABA itself as bait. Many antagonists and agonists have stronger binding capacity than GABA (Çiçek et al., 2018). Therefore, in the future we will use other GABA receptor regulators and GABA homologues as bait to identify GABA-binding proteins in plants via affinity chromatography.

## Acknowledgments

We would like to express our gratitude to Professor Jian-Feng Liu (University of Hua Zhong Science and Technology, China) for kindly providing the photo-affinity probes, probeR and probeC. We would also like to thank Professor Xiao-Dong Li (Wuhan University, China) for kindly providing the rat brain tissue sections. This work was supported by the “973” and National Basic Research Program of China (2013CB126900; 2013CB945100).

## Author contributions

J.Z. designed and conducted the probe detection experiments and magnetic affinity chromatography; J.Z. and J.Z.G. synthesized the GABA-coupled magnetic beads; S.S.L. isolated the pollen protoplasts and analyzed the images; J.Z. and S.S.L. wrote the initial version of the manuscript.
